# Induced sputum metabolomic profiles and oxidative stress are associated with chronic obstructive pulmonary disease (COPD) severity: potential use for predictive, preventive, and personalized medicine

**DOI:** 10.1007/s13167-020-00227-w

**Published:** 2020-11-04

**Authors:** Tao Zhu, Shanqun Li, Jiajia Wang, Chunfang Liu, Lei Gao, Yuzhen Zeng, Ruolin Mao, Bo Cui, Hong Ji, Zhihong Chen

**Affiliations:** 1 Respiratory Medicine, Second Affiliated Hospital of Chongqing Medical University, Chongqing, 400010 China; 2 Department of Respiratory and Critical Care Medicine, Zhongshan Hospital of Fudan University, Shanghai, 20032 China; 3 Rheumatology Medicine, Second Affiliated Hospital of Chongqing Medical University, Chongqing, 400010 China; 4 California National Primate Research Center, and Department of Anatomy, Physiology & Cell Biology, School of Veterinary Medicine, University of California, Davis, CA 95616 USA

**Keywords:** Induced sputum, Chronic obstructive pulmonary disease (COPD), Lung function, Metabolomics, Glycerophospholipid metabolism pathway, Predictive preventive personalized medicine (PPPM)

## Abstract

Chronic obstructive pulmonary disease (COPD) is a highly heterogeneous disease, and metabolomics plays a hub role in predictive, preventive, and personalized medicine (PPPM) related to COPD. This study thus aimed to reveal the role of induced sputum metabolomics in predicting COPD severity. In this pilot study, a total of 20 COPD patients were included. The induced sputum metabolites were assayed using a liquid chromatography-mass spectrometry (LC-MS/MS) system. Five oxidative stress products (myeloperoxidase (MPO), superoxide dismutase (SOD), glutathione (GSH), neutrophil elastase (NE), and 8-iso-PGF2α) in induced sputum were measured by ELISA, and the metabolomic profiles were distinguished by principal component analysis (PCA) and orthogonal projections to latent structures discriminant analysis (OPLS-DA). The Kyoto Encyclopedia of Genes and Genomes (KEGG) was used for pathway enrichment analysis, and a significant difference in induced sputum metabolomics was observed between moderate and severe COPD. The KEGG analysis revealed that the glycerophospholipid metabolism pathway was downregulated in severe COPD. Due to the critical role of glycerophospholipid metabolism in oxidative stress, significant negative correlations were discovered between glycerophospholipid metabolites and three oxidative stress products (SOD, MPO, and 8-iso-PGF2α). The diagnostic values of SOD, MPO, and 8-iso-PGF2α in induced sputum were found to exhibit high sensitivities and specificities in the prediction of COPD severity. Collectively, this study provides the first identification of the association between induced sputum metabolomic profiles and COPD severity, indicating the potential value of metabolomics in PPPM for COPD management. The study also reveals the correlation between glycerophospholipid metabolites and oxidative stress products and their value for predicting COPD severity.

**Supplementary Information:**

The online version contains supplementary material available at 10.1007/s13167-020-00227-w.

## Introduction

Chronic obstructive pulmonary disease (COPD), the most common pulmonary disease worldwide, is characterized by persistent airflow limitation and incompletely reversible airway construction. The global prevalence of COPD was approximately 11.7% (95% CI 8.4%–15.0%) in 2010 [1, 2], and it has been estimated that COPD leads to more than 3 million deaths each year [1–3]. COPD is considered a highly heterogeneous disease with multiple phenotypes and endotypes [2, 4]. According to the Global Initiative for Chronic Obstructive Lung Disease (GOLD), the combination of the current severity of symptoms and a history of acute exacerbations within the previous year is recommended for assessing the phenotypes, which are divided into four different groups (A, B, C, and D), and for guiding the management of patients with COPD [2]. Moreover, the assessment of different aspects of COPD has identified a number of biomarkers [5]. For instance, the eosinophil percentage in the blood is a good biomarker for predicting the response to inhaled corticosteroids and the risk of readmission [6–8]. Nevertheless, substantial heterogeneities in clinical features, the treatment response, and prognosis have also been observed in a certain subgroup of patients with COPD [9–11]. Thus, there is an urgent need to move the management of COPD from the current “one-size-fits-all” approach to predictive, preventive, and personalized medicine (PPPM).

An imbalance in metabolic homeostasis is essential for the pathogenesis and progression of many diseases, such as cancers, diabetes, suboptimal health status (SHS), and COPD [12–21]. Mounting evidence supports the notion that metabolomics plays a hub role in the PPPM approach for different aspects of COPD [11, 12, 21–23]. Moreover, recent studies have shown that differential metabolomic profiles are associated with the COPD status in both patients and animals [22, 24–26]. Cruickshank-Quinn et al. reported that 2999 differential metabolites in plasma are associated with COPD outcomes, including the lung function, exacerbation frequency, degree of emphysema, and bronchodilator response (BDR) [22]. Pinto-Plata V et al. identified 79 differential plasma metabolites between surviving and nonsurviving COPD patients [25], and Fang W et al. found that 32 differential metabolites in plasma are associated with the COPD status in rats [26]. Therefore, a comprehensive understanding of metabolomic profiles might provide insights for the PPPM strategy for COPD.

Induced sputum is a commonly used method for differentiating the phenotypes and inflammatory endotypes of COPD and asthma. Compared with plasma, induced sputum is more relevant to pathological alterations in the lower airway with less confounders in the results. Moreover, induced sputum is less contaminated and yields more accurate results than spontaneous sputum [27, 28]. Therefore, induced sputum is considered a material for the effective and accurate assessment of airway diseases that exhibits high safety and tolerability in clinical practice. The primary aims of this study were to reveal the role of the metabolomic profiles of induced sputum in the determination of COPD severity and to explore the metabolic biomarkers in induced sputum that can predict COPD severity.

## Material and methods

### Study design and population

This pilot study was performed at the Respiratory Department of Zhongshan Hospital of Fudan University from January 2017 to December 2018. This study was approved by the Research Ethics Committees of Zhongshan Hospital of Fudan University (No. B2014-108) in accordance with the Declaration of Helsinki. Informed consent was obtained from all the patients by the responsible physician or an appropriately trained staff member. Standard care and treatments were provided according to the current clinical guidelines [2, 29].

### Inclusion and exclusion criteria

**Fig. 1:**
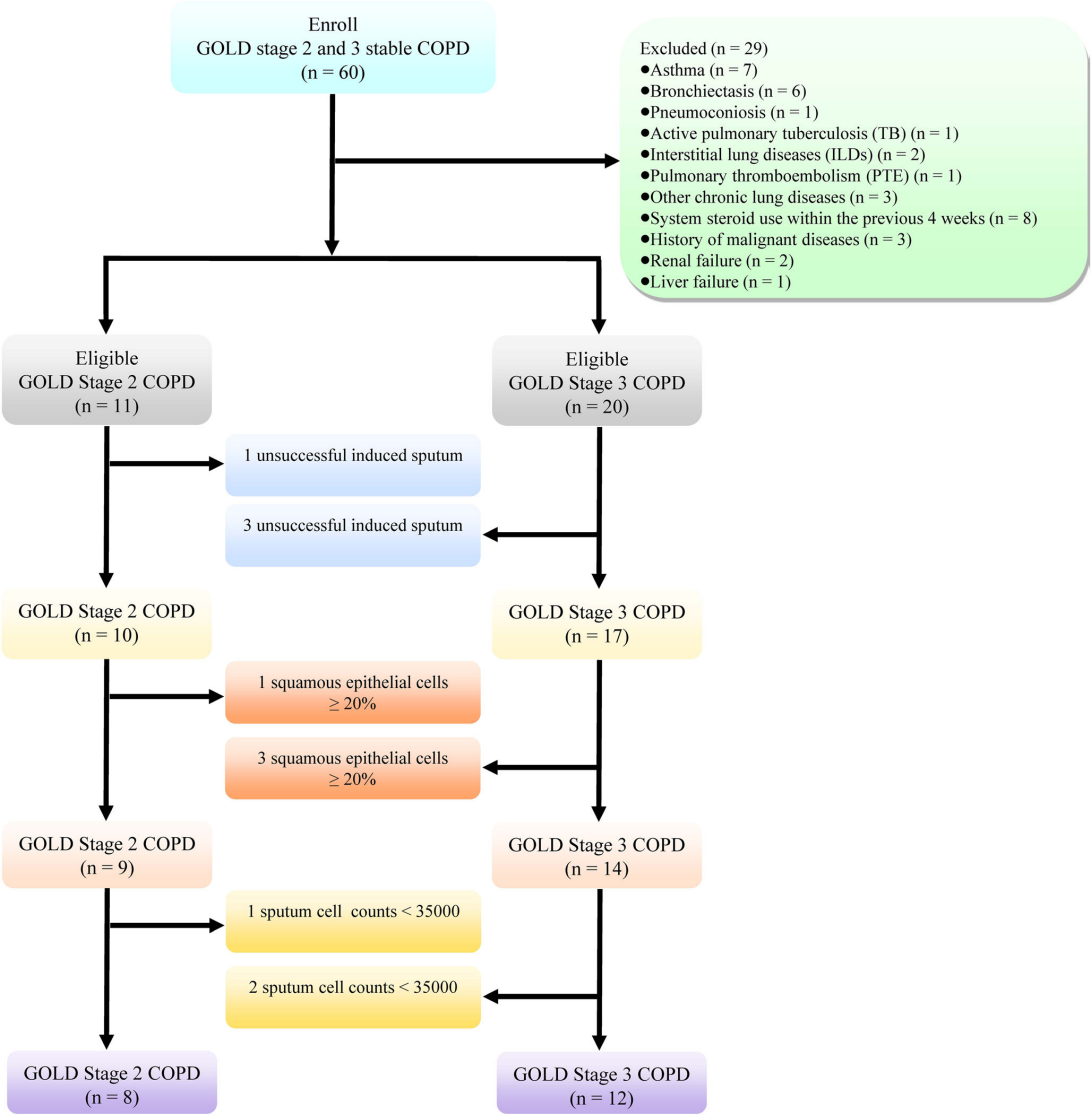
Flow diagram of the study

The inclusion criterion was stable COPD with a lung function classified as Global Initiative for Chronic Obstructive Lung Disease (GOLD) stage 2 (50% ≤ forced expiratory volume in 1st second % (FEV1%) < 80%) or 3 (30% ≤ FEV1% < 50%) [2]. Additionally, FEV1 was defined as the maximum amount of air that the subject can forcibly expel during the 1st second following maximal inhalation [30]. The exclusion criteria were as follows: age < 40 years, active pulmonary tuberculosis (TB), asthma, bronchiectasis, pneumoconiosis, interstitial lung diseases (ILDs), pulmonary thromboembolism (PTE), other chronic lung diseases, systemic steroid use within the previous 4 weeks, history of malignant diseases, renal failure, and liver failure. A total of 60 patients with stable COPD at GOLD stages 2 and 3 were recruited, and 29 were excluded based on the abovementioned criteria. Furthermore, four patients were unable to complete or tolerate sputum induction. The induced sputum from seven patients was disqualified (the sputum samples from four patients had ≥ 20% squamous epithelial cells, and the sputum samples from three patients had cell counts < 3500) [31]. In the end, 20 patients with qualified induced sputum samples were included: eight patients with COPD at GOLD stage 2 (moderate) and 12 patients with COPD at GOLD stage 3 (severe) (Fig. 1).

### Clinical data collection

In our study, demographic data, underlying diseases, comorbidities, modified Medical Research Council dyspnea scale (mMRC) scores, and inhalation therapy information were recorded and collected. Routine blood, erythrocyte sedimentation rate (ESR), C-reactive protein (CRP), and lung function tests were performed on the same day. To ensure the accuracy of the diagnosis and exclude most other lung diseases, all the patients underwent a high-resolution CT (HRCT) scan (64-channel CT machines with 64 × 0.625-mm collimation, 1.00-mm slice thickness, 1.00-mm intervals, reconstruction with standard algorithm, 120 kVp, and 90 mAs) within 24 h after admission. The radiation dose of HRCT was in the safe range [32, 33]. All CT results were reviewed by one independent radiologist and one pulmonologist at the hospital.

### Induced sputum collection

Prior to nebulization, the enrolled subjects drank 500 mL of water and gargled. Before hypertonic saline induction, 400 μg of salbutamol (Ventolin, GSK, UK) was inhaled. The participants were then nebulized using an ultrasonic atomizer (DeVilbiss Healthcare, Australia) with 3% NaCl for 7 min. After a short rest period, the abovementioned procedures were repeated up to three times. All phlegm clots with visibly greater solidity were carefully selected and placed in a preweighed Eppendorf tube (Eppendorf Corp. Germany). Phlegm clots less than 0.02 g indicated sputum induction failure. Phlegm clots greater than 0.02 g were processed as follows. The phlegm clots were diluted 4 times with a mixture of 0.05% dithiothreitol (Sputolysin; Calbiochem Corp., San Diego, CA, USA) and Dulbecco’s PBS (ThermoFisher Corp., USA). The mixture was placed into a shaker at room temperature (RT) for 30 min until the sputum mass dissolved. The mixture of sputum and Sputolysin was passed through a nylon filter apparatus. The filtrate was collected and centrifuged at 1000 g and 4 °C for 4 min. The supernatant was collected and stored in liquid nitrogen, and the cell pellet was resuspended in PBS. Qualification of the induced sputum sample was checked by H&E staining. The samples with a squamous cell percentage ≤ 20% and/or sputum cell count > 3500 satisfied the test criteria, and the other (unqualified) samples were not subjected to further processed [31]. Subsequently, the cell sediments were resuspended in 1400 μL of quencher solution (including 200 μL of 0.085% ammonium bicarbonate (Ambic) and 1200 μL of 60% methanol). The samples were then centrifuged at 1000 g and 4 °C for 4 min, and the cell pellets and supernatants were stored in liquid nitrogen for further analyses [34].

### Identification of metabolites in induced sputum

According to previous reports and the manufacturer’s instructions, the metabolites of cells in induced sputum were assayed using an Q Exactive Orbitrap LC-MS/MS system [35–37]. Biotree Biotech Co., Ltd. (Shanghai, China) helped with the LC-MS analysis.

First, the cell pellet was dried under a gentle nitrogen flow, and 1000 μL of extracted solution (acetonitrile:methanol:water = 2:2:1) with 1 μg/mL internal standard was added to the sample. After vortexing for 30 s, the samples were homogenized at 35 Hz for 4 min and sonicated for 5 min in an ice-water bath. The homogenization-and-sonication cycle was repeated three times. The samples were then incubated at − 20 °C for 1 h and centrifuged at 11000 rpm and 4 °C for 15 min. The resulting supernatant was transferred to a fresh glass vial for LC-MS analysis. Moreover, a mixture from various samples (10 μL) was used as a quality control (QC) sample. Subsequently, a 1290 Infinity series UHPLC System (Agilent Technologies, CA, USA) coupled with a UPLC Waters ACQUITY UPLC HSS T3 (2.1 mm × 100 mm, 1.8 μm) and an Q exactive mass spectrometer (Thermo) was used to identify and analyze the metabolites in induced sputum. Mobile phase A consisted of 0.1% formic acid in water in the positive mode and 5 mmol/L ammonium acetate in water in the negative mode, and mobile phase B was acetonitrile. The elution gradient was set as follows: 0~1.0 min, 1% B; 1.0~8.0 min, 1~99% B; 8.0~10.0 min, 99% B; 10.0~10.1 min, 99~1% B; and 10.1~ 12 min, 1% B. The flow rate was 0.5 mL/min, and the injected volume was 2 μL. The QE mass spectrometer was used for its ability to acquire MS/MS spectra in the data-dependent acquisition (DDA) mode, and this process was controlled by the acquisition software (Xcalibur 4.0.27, Thermo). In this mode, the acquisition software continuously evaluates the full-scan MS spectrum. The ESI source conditions were set as follows: sheath gas flow rate of 45 Arb, Aux gas flow rate of 15 Arb, capillary temperature of 400 °C, full MS resolution of 70,000, MS/MS resolution of 17,500, collision energy of 20/40/60 eV in the normalized collisional energy (NCE) mode, and spray voltage of 4.0 kV (positive) or − 3.6 kV (negative). The injection volume was 1 μL. The MS/MS spectra were measured with the Triple TOF mass spectrometer on a data-dependent acquisition (DDA) in the LC-MS experiment. Meanwhile, top 3 intensive ions (peaks) in each MS1 scan were selected for MS/MS analysis.

### Levels of myeloperoxidase, superoxide dismutase, glutathione, neutrophil elastase, and 8-iso-PGF2α in induced sputum

According to our previous studies [38–41] and the manufacturer’s instructions, the levels of myeloperoxidase (MPO), superoxide dismutase (SOD), glutathione (GSH), neutrophil elastase (NE), and 8-iso-PGF2α in the supernatant of induced sputum were measured by ELISA (R&D Systems, Minneapolis, MN, USA). In brief, a 96-well microplate was coated with 100 μL of the capture antibodies (MPO, SOD, GSH, NE, or 8-iso-PGF2α) overnight at 4 °C. Unbound capture antibody was blocked at RT for 1 h, and 100 μL of induced sputum supernatant was added to each well of the microplate for incubation at RT for 2 h. The detection antibody was added, and the plate was incubated at RT for 2 h. Between each step, the plate was washed with a detergent solution to remove any nonspecifically bound proteins or antibodies. Subsequently, 50 μL of termination solution was added to each well, and the absorbance was read using a microplate reader at 450 nm [38].

### Statistical analysis

The data were entered into a computer spreadsheet program (Microsoft Office Excel 2010) by designated staff members. All the analyses were performed using SPSS 22.0. The baseline characteristics of the study population are described. Continuous variables are expressed as the means ± standard deviations (SDs), and categorical data are expressed as frequencies. Non-normally distributed continuous data are presented as the medians and interquartile ranges (IQRs). The distribution of categorical groups was examined using the Kolmogorov-Smirnov test. The categorical variables were analyzed using the chi-square test, and the continuous variables were analyzed by Student’s *t* test. Ordinal variables and continuous variables without a normal distribution were analyzed with the Mann-Whitney U test. The Spearman rank correlation coefficient was used for the correlation analyses. A threshold of *P* < 0.05 was considered significant.

For the metabolomics analysis, mass spectrometry (MS) raw data files were converted to mzXML format using ProteoWizard software (version 3.0.19282) and processed with R software package XCMS (version 3.2) for peak detection, extraction, alignment, and integration [35, 42, 43]. The peaks were detected, and metabolites could be identified through the interquartile range denoising method. The missing values in the raw data were filled by half of the minimum value. Additionally, the overall normalization method was used for data analysis. To make the metabolomics data reproducible, the relative standard derivation (RSD) of the peaks in the QC samples larger than 30% were filtered out. The multivariate analysis, principal component analysis (PCA), and orthogonal projections to latent structure discriminant analysis (OPLS-DA) were performed using SIMCA software (v14.1; Sartorius Stedim Data Analytics AB, Umea, Sweden). PCA was used to show the distribution of the original data. OPLS-DA was used to further observe the separation between two groups and to further understand the variables responsible for classification. The variable importance in the projection (VIP) of the first principal component obtained in the OPLS-DA analysis was obtained. The metabolites with a VIP value > 1 in the OPLS-DA analysis and a *P* value < 0.05 in the univariate analysis were considered significantly different. Moreover, the OPLS-DA model quality was evaluated with standard parameters (R2Y and Q2) by 200 permutations. Additionally, the data were matched using BiotreeDB (V2.1) to establish a secondary mass spectrometry database for material annotation [44]. The commercial database KEGG (http://www.genome.jp/kegg/) was used for pathway analysis.

## Results

### Demographic characteristics and laboratory parameters of patients with COPD

A total of 60 patients were initially enrolled in this study, and in the end, eight patients with moderate (GOLD stage 2) COPD and 12 patients with severe (GOLD stage 3) COPD from whom qualified induced sputum samples were obtained were included in this study (Fig. 1). The FEV1% and FEV1/FVC% values of the patients with severe COPD were noticeably lower than those of the patients with moderate COPD (Table 1). No differences in age, sex, smoking status, BMI, mMRC scores, inhalation therapies, underlying diseases, or laboratory parameters were found between the patients with moderate COPD and those with severe COPD.

**Table 1:** The demographic data and laboratory findings from COPD patients (*n* = 20)

	Moderate COPD (*n* = 8)	Severe COPD (*n* = 12)	Statistical values	*P*
Sex (male, (%))	8 (100.00%)	11 (91.67%)	0.702	0.402
Age (years)	67.5000 ± 8.60233	67.0000 ± 5.30866	0.162	0.873
Body mass index (BMI)	21.6350 ± 2.41780	21.8017 ± 3.10319	− 0.128	0.900
Smoking			0.208	0.901
Non-smoking	2	2		
Ex-smoking	3	5		
Current-smoking	3	5		
mMRC scores	1.2500 ± 0.70711	1.7500 ± 0.86603	− 1.356	0.192
Lung functions				
Forced expiratory volume in 1st second % (FEV1%)	62.2250 ± 8.02064	40.3500 ± 7.24061	6.345	0.000*
Forced expiratory volume in 1st second/forced vital capacity % (FEV1/FVC%)	60.6788 ± 8.07667	47.5508 ± 9.40506	3.227	0.005*
Underlying diseases/comorbidities				
Cor pulmonale	1	1	0.093	0.761
Pulmonary hypertension (PH)	0	1	0.702	0.402
Coronary artery disease (CAD)	2	3	0.000	1.000
Hypertension	4	5	0.135	0.714
Type 2 diabetes (T2DM)	2	3	0.000	1.000
Atrial fibrillation (Af)	0	1	0.702	0.402
Inhalation therapy			0.104	0.949
ICS/LABA	3	5		
LAMA	4	6		
ICS/LABA/LAMA	1	1		
Laboratory parameters				
White blood cells (WBC) (× 10^9^/L)	6.4988 ± 2.12200	6.8625 ± 1.41555	− 0.462	0.650
Neutrophils (NS) (× 10^9^/L)	4.2525 ± 1.98737	4.7558 ± 1.20220	− 0.709	0.487
Lymphocytes (× 10^9^/L)	1.5988 ± 0.66398	1.4308 ± 0.42374	0.694	0.497
Eosinophils (EOS) (× 10^9^/L)	0.2275 ± 0.23236	0.1833 ± 0.10369	0.583	0.567
Neutrophils-to-lymphocytes ratio (NLR)	3.1438 ± 2.24662	3.5950 ± 1.34660	− 0.564	0.580
NS%	63.6275 ± 11.04820	68.9108 ± 6.29898	− 1.367	0.189
EOS%	3.4962 ± 3.2007	2.7450 ± 1.61699	0.697	0.495
Lymphocytes%	26.4613 ± 10.58444	21.0542 ± 5.78485	1.481	0.156
C-reactive protein (CRP) (mg/mL)	8.5750 ± 10.11163	20.0750 ± 14.78796	− 1.913	0.072
Erythrocyte sedimentation rate (ESR) (mm/first hour)	14.0000 ± 14.10167	15.6667 ± 17.55166	− 0.224	0.825
Red blood cells (RBC) (× 10^12^/L)	4.2875 ± 0.59122	4.5325 ± 0.24893	− 1.288	0.214
Hemoglobin (Hb) (g/L)	133.7500 ± 15.42493	139.3333 ± 7.31541	− 1.093	0.289
Platelets (PLT) (× 10^9^/L)	161.0000 ± 92.48784	204.4167 ± 60.20036	− 1.278	0.218

*P value < 0.05

### Identification and quantification of LC-MS/MS system compounds

The ionization source for the LC-MS/MS was electrospray ionization, including the positive (POS) and negative (NEG) ion modes, and a total of 10,503 POS peaks and 4958 NEG peaks were found after data preprocessing (Supplementary Table S1).

### Multivariate analysis of metabolites

**Fig. 2:**
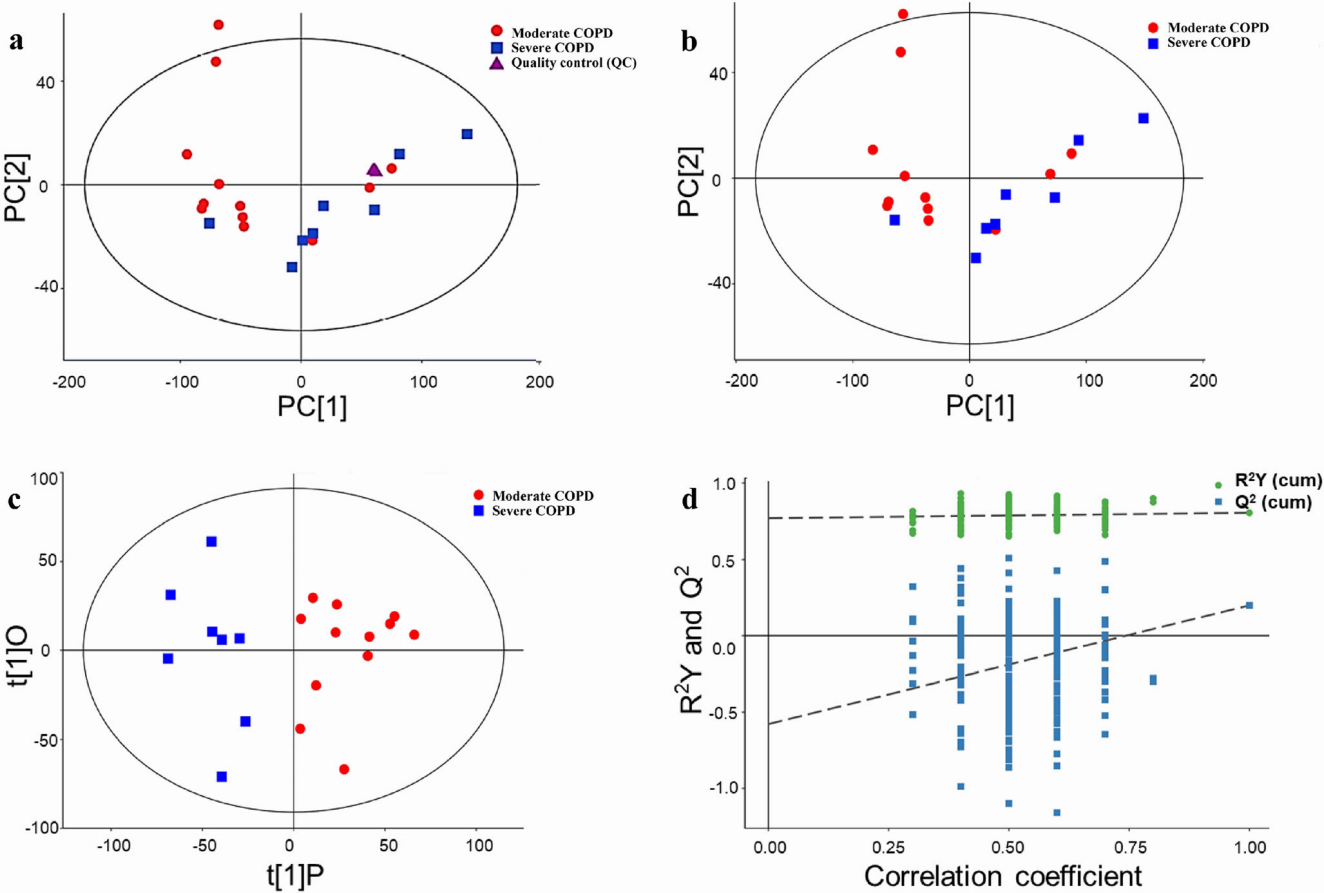
PCA score plots, OPLS-DA score plot, and corresponding validation plot of OPLS-DA results derived from the metabolomics profiles of induced sputum between moderate and severe COPD. **a** PCA score plot with four quality controls (QC). **b** PCA score plot without the QC samples. **c** OPLS-DA score plot. **d** Permutation test (*n* = 200) of the OPLS-DA model

Multivariate analyses, namely, PCA and OPLS-DA, were performed to comprehensively compare the induced sputum metabolomic profiles between moderate and severe COPD and to reveal the degree of diversity between the two groups. Four quality control (QC) samples were included to evaluate the stability and repeatability of the system, and the QCs were clustered together and separated from the samples from the study subjects, which indicated the correctness of the PCA (Fig. 2a). The PCA score plot presented clear differences between moderate and severe COPD, and one outlier was located beyond 95% Hotelling’s *T*-squared ellipse (Fig. 2b). As shown in Fig. 2b, most moderate COPD samples clustered on the left, whereas all severe COPD clustered on the right. However, three moderate COPD samples located on the right were also observed in the PCA score plots.

An OPLS-DA was performed to further compare the induced sputum metabolomic profiles between moderate and severe COPD. The OPLS-DA score plot showed significant differences in the induced sputum metabolomic profiles between the moderate and severe COPD samples (Fig. 2c). As shown in Fig. 2c, all moderate COPD samples clustered on the right, whereas all severe COPD clustered on the left, and no overlap was observed. This result indicated the existence of significant differences in the induced sputum metabolomic profiles between patients with moderate COPD and those with severe COPD. Additionally, the permutation test yielded *R*^2^*Y* (cum) and *Q*^2^ (cum) values of 0.77 and − 0.58, respectively, and these results indicated the lack of overfitting and the good predictive ability of the OPLS-DA model (Fig. 2d), which indicated the suitability of the model for subsequent optimization analyses.

### Identification of significantly different metabolites and pathways associated with COPD severity

In our study, 573 positive-ion-mode (POS) metabolites with VIP values > 1 in the OPLS-DA analysis and *P* values < 0.05 in the univariate analysis were found between moderate and severe COPD samples (Supplementary Table S2), which indicated that these 573 POS metabolites were associated with COPD severity. Among these metabolites, 237 were mass spectrometry 1 (MS1) metabolites, 21 were mass spectrometry 2 (MS2) metabolites, and 315 were “unknown” metabolites based on the BiotreeDB database. However, no negative-ion-mode (NEG) metabolites associated with COPD severity were found.

**Fig. 3:**
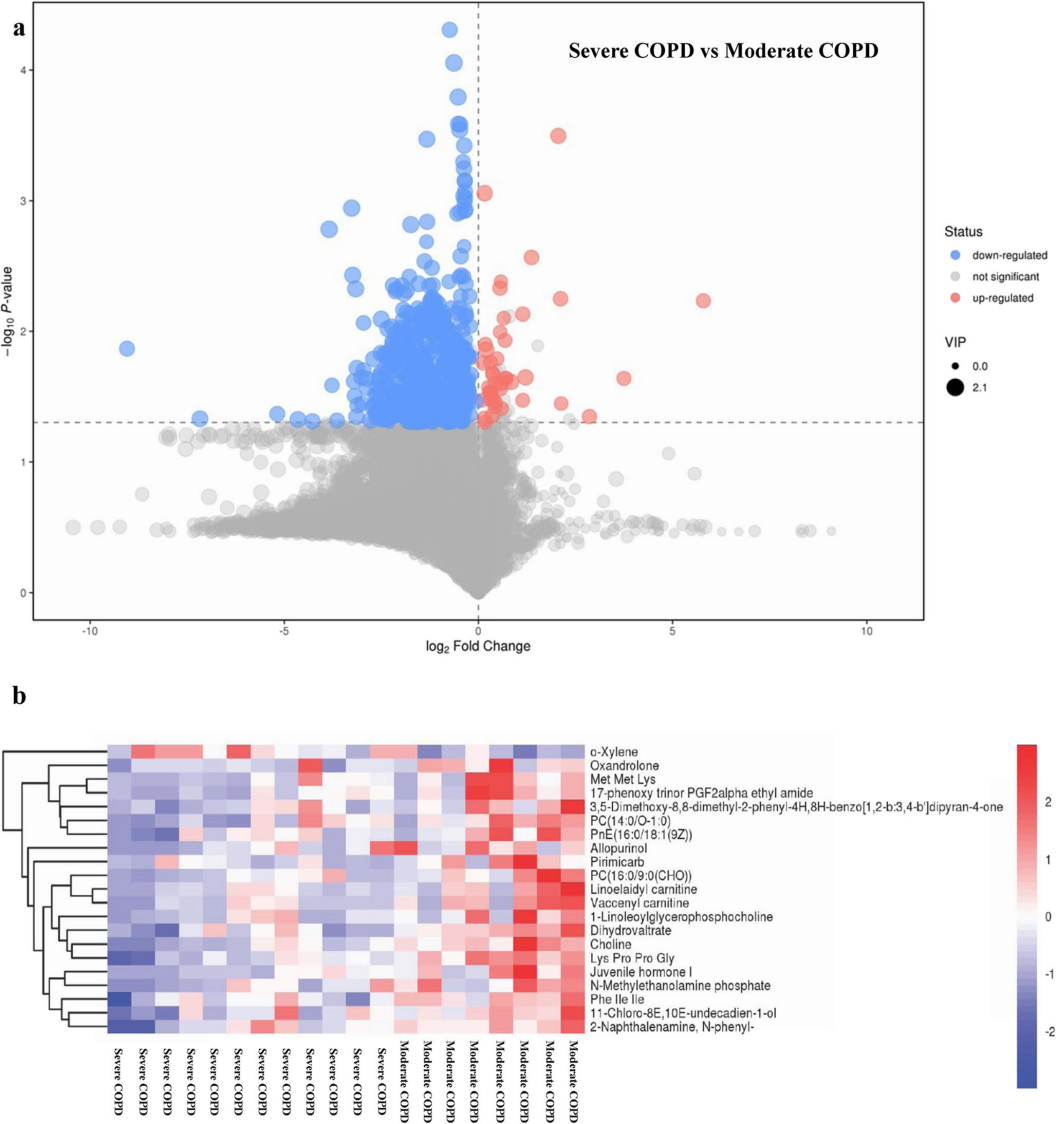
Identification of the differential metabolomics profiles of induced sputum between moderate and severe COPD based on a volcano plot and hierarchical clustering analysis. **a** Volcano plot. The downregulated and upregulated metabolites in severe compared with moderate COPD are marked in blue and red, respectively. *X*-axis: log_2_ fold change of metabolites; *Y*-axis: fold change of –log_10_ *P* value determined by Student’s *t* test. The dot size represents the variable importance in the projection (VIP) value. **b** Heatmap of the hierarchical clustering analysis. Twenty-one MS2 differential metabolites are presented

Specifically, 42 metabolites were increased and 531 metabolites were reduced in severe COPD compared with moderate COPD (Supplementary Table S2). These 573 POS metabolites associated with COPD severity were then summarized in a volcano plot (Fig. 3a): the 42 metabolites (red spots) increased in severe compared with moderate COPD were located on the right, and the 531 reduced metabolites (blue spots) were located on the left (Fig. 3a). In addition, 1 and 20 of the 21 MS2 metabolites was found at higher and lower levels, respectively, in severe compared with moderate COPD (Supplementary Table S2). Furthermore, a heatmap was used to classify the upregulated and downregulated MS2 metabolites in patients with severe COPD compared with those with moderate COPD (Fig. 3b).

Additionally, a KEGG pathway analysis was performed to explore the enriched metabolic pathways associated with COPD severity. The results showed that the glycerophospholipid metabolism pathway was the only enriched metabolic pathway associated with COPD severity, and this pathway was downregulated in severe compared with moderate COPD (Supplementary Table S2).

### Predictive value of five oxidative stress products in induced sputum for predicting COPD severity

**Fig. 4:**
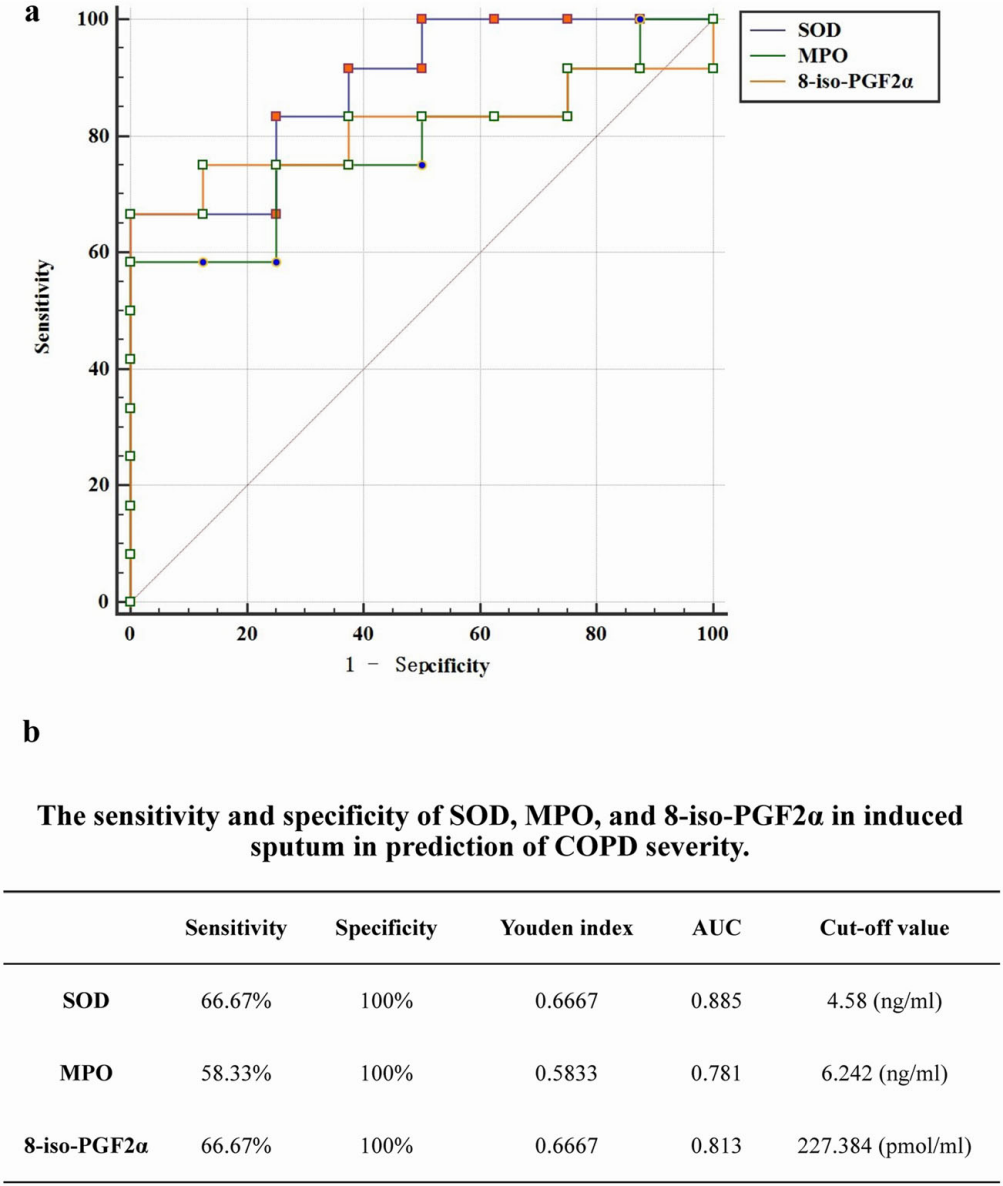
Values of the SOD, MPO, and 8-iso-PGF2α levels in induced sputum for predicting COPD severity. **a** ROC curves. **b** Sensitivity, specificity, Youden index, AUC, and cutoff value

Because the glycerophospholipid metabolism pathway is essential for oxidative stress [45–47], the levels of five oxidative stress products (MPO, SOD, GSH, NE, and 8-iso-PGF2α) in induced sputum were measured by ELISA to evaluate the association between oxidative stress and COPD severity. The levels of SOD, MPO, and 8-iso-PGF2α in the induced sputum samples from the patients with severe COPD were significantly higher than those in the samples from the patients with moderate COPD (Table 2). No differences in GSH and NE were observed between moderate and severe COPD. Moreover, the levels of SOD (AUC = 0.885), MPO (AUC = 0.781), and 8-iso-PGF2α (AUC = 0.813) in induced sputum showed high sensitivities and specificities for the prediction of COPD severity (Fig. 4).

**Table 2:** The levels of SOD, MPO, GSH, NE, and 8-iso-PGF2α in induced sputum in COPD patients (*n* = 20)

	Moderate COPD (*n* = 8)	Severe COPD (*n* = 12)	Statistical values	P
SOD (ng/mL)	2.81702750 ± 1.481169877	5.87841667 ± 1.684597522	− 4.170	0.001*
MPO (ng/mL)	5.20834875 ± 0.849961544	7.11504083 ± 2.127440318	− 2.393	0.028*
GSH (pg/mL)	48.89454875 ± 81.860376895	59.50663000 ± 100.387792570	− 0.248	0.807
NE (pg/mL)	275.44425000 ± 253.353683063	250.36500000 ± 262.347043724	0.212	0.834
8-iso-PGF2α (pmol/mL)	131.84655462 ± 50.691770294	343.47142350 ± 226.995290954	− 2.572	0.019*

*P value < 0.05

### Correlations between glycerophospholipids (choline, *N*-methylethanolamine phosphate, and 1-linoleoylglycerophosphocholine) and levels of oxidative stress products in induced sputum from patients with COPD

The correlations between glycerophospholipids (choline, *N*-methylethanolamine phosphate (NMethy), and 1-linoleoylglycerophosphocholine (1-LGPC)) and the levels of five oxidative stress products in induced sputum from patients with stable COPD were explored by Spearman correlation analysis. As shown in Table 3, SOD was significantly negatively correlated with three glycerophospholipids (choline, NMethy, and 1-LGPC), and 1-LGPC was markedly negatively correlated with MPO and 8-iso-PGF2α.

**Table 3:** The correlations between lung function and 3 glycerophospholipids and induced sputum 5 oxidative stress products in COPD patients (*n* = 20)

		SOD	MPO	GSH	NE	8-iso-PGF2α
FEV1%	R	− 0.405	− 0.432	0.171	− 0.174	− 0.606
	P	0.077	0.057	0.470	0.462	0.005*
FEV1/FVC%	R	− 0.313	− 0.512	0.275	− 0.217	− 0.705
	P	0.179	0.021*	0.240	0.359	0.001*
Choline	R	− 0.650	− 0.409	− 0.185	0.117	− 0.411
	P	0.002*	0.073	0.435	0.622	0.072
NMethy	R	− 0.653	− 0.421	− 0.188	− 0.200	− 0.259
	P	0.002*	0.064	0.427	0.398	0.271
1-Linoleoylglycerophosphocholine (1-LGPC)	R	− 0.489	− 0.514	− 0.132	− 0.281	− 0.504
	P	0.029*	0.021*	0.578	0.230	0.024*

*P value < 0.05

### Correlations between lung function and SOD, MPO, GSH, NE, and 8-iso-PGF2α levels in induced sputum of COPD patients

Because COPD severity is defined by the degree of lung function impairments [2], the correlations between lung function (FEV1% and FEV1/FVC%) and five oxidative stress products in induced sputum from patients with stable COPD were explored by Spearman correlation analysis. Significant negative correlations were observed between FEV1% and the 8-iso-PGF2α level and between FEV1/FVC% and the MPO and 8-iso-PGF2α levels (Table 3).

## Discussion

This pilot study provides the first demonstration of an association between the metabolomics profile of induced sputum and COPD severity, and the results indicate the potential value of induced sputum metabolomics in the PPPM-based management of COPD. Simultaneously, a subset of COPD severity–associated metabolites were identified, and these were found to be promising biomarkers for predicting COPD severity. In this study, we found that 573 POS differential metabolites (DMs), including 237 MS1, 21 MS2, and 315 “unknown” metabolites, were associated with lung function impairments (GOLD stages) in patients with stable COPD. Among the 573 DMs, 42 and 531 metabolites were found at higher and lower levels, respectively, in patients with severe COPD. Moreover, among the 21 MS2 metabolites, 1 and 20 metabolites were increased and reduced, respectively, in severe compared with moderate COPD. We then found that the glycerophospholipid metabolism pathway was associated with COPD severity. Additionally, three oxidative stress products (MPO, SOD, and 8-iso-PGF2α) in induced sputum that exhibited high sensitivities and specificities for the prediction of COPD severity were identified. We also found that the MPO and 8-iso-PGF2α levels in induced sputum were negatively correlated with lung function and that the SOD, MPO, and 8-iso-PGF2α levels in induced sputum were negatively correlated with glycerophospholipids (choline, NMethy, and 1-LGPC) in patients with stable COPD.

The prevalence of COPD has continued to increase in recent decades [2], and it has been estimated that COPD will become the third leading cause of disease-induced mortality worldwide by 2025 to 2030 [1, 2]. In addition, COPD is a highly heterogeneous disease [11]. Significant individual differences in many variables, including risk factors, clinical features, comorbidities, and therapeutic responses to prognosis, have been observed among patients with COPD [2]. From one point of view, the consideration of COPD as a single disease is less appropriate because COPD is probably a syndrome that encompasses several obstructive airway disorders that share a common exposure but differ in terms of the mechanism of the disease and the response to treatment [48, 49]. Therefore, predictive, preventive, and personalized medicine (PPPM)–based management approaches are urgently needed to guide the diagnosis, severity assessment, therapeutic options, and prognosis of COPD. Accumulating evidence obtained in recent studies reveals that abnormal metabolism plays a key role in the pathogenesis of COPD [12, 24, 50]. Compared with traditional metabolic study methods, metabolomics tools provide insight into underlying relationships among metabolites, and these findings allows researchers to obtain a complete picture of the role of metabolites in the pathogenesis of diseases. Moreover, several studies have shown that metabolomics is an ideal and promising method for evaluating the phenotype, severity, and individual differences among patients with COPD, which indicates that metabolomics is an essential tool for PPPM in COPD [12, 50]. Several studies have found distinct differences in the metabolomic profile between patients with COPD and without COPD, and similar findings have been obtained in humans and animals [24, 26, 42, 50, 51]. Fang et al. showed that 32 dysregulated metabolites in plasma are associated with the COPD status in rats [26]. Van der Does et al. revealed that the levels of free alpha-linolenic acid, linoleic acid, eicosapentaenoic acid (EPA), omega-3, and EPA- and docosahexaenoic acid-derived oxylipins in the sputum of patients with COPD are noticeably lower than those in the sputum of healthy subjects [36]. These researchers also noticed that the levels of free arachidonic acid and docosapentaenoic acid in sputum were significantly increased in acute exacerbation of COPD (AECOPD) compared with stable COPD. Fortis et al. showed that both the serum and urine metabolomic profiles showed marked differences between patients with stable COPD and those with AECOPD [42]. Additionally, Ghosh et al. revealed that 12 differential metabolites in serum were dysregulated in patients with asthma-COPD overlap (ACO) compared with both patients with asthma and patients with COPD [51]. However, the association between the metabolomic profiles of induced sputum and COPD severity has not been previously studied. This study aimed to explore the differential metabolites in induced sputum between patients with moderate (GOLD stage 2) and severe (GOLD stage 3) stable COPD. In our study, 60 patients were enrolled, and 20 patients with qualified induced sputum samples were included: 8 with moderate (GOLD stage 2) COPD and 12 with severe (GOLD stage 3) COPD (Fig. 1). No differences in sex, age, BMI, smoking status, underlying diseases, inhalation therapies, complications, or laboratory parameters were found between these two groups. These results suggested that demographic characteristics, treatments, and included laboratory parameters are not associated with the severity of stable COPD, and these data also indicated that the internal validity of the study was good.

Induced sputum is a direct, reliable, sensitive, simple, and repeatable approach for evaluating inflammatory phenotypes and severity and for studying the pathogenesis of COPD and other pulmonary diseases, such as asthma and interstitial lung diseases (ILDs) [52–57]. In our study, the metabolites in induced sputum were assayed using a Q Exactive Orbitrap LC-MS/MS system. The results showed that 573 POS differential metabolites were associated with COPD severity, and among these, 237 were MS1 metabolites, 21 were MS2 metabolites, and 315 were “unknown” metabolites. However, no COPD severity–associated NEG metabolites were detected. Furthermore, a KEGG pathway analysis revealed that the glycerophospholipid metabolism pathway was the only enriched metabolic pathway associated with COPD severity. Glycerophospholipids, which are the most abundant phospholipids in the lungs [58], are critical for the synthesis of pulmonary surfactant (PS), lung development, oxidative stress, lung defense, and the inflammatory response [58–60]. Xu et al. found that both the glycerophospholipids levels in both serum and the bronchoalveolar lavage fluid (BALF) are upregulated in rats with LPS-induced acute lung injury (ALI) [58]. In addition, some studies have revealed that the glycerophospholipid metabolism pathway is involved in COPD [22, 26, 61]. Telenga et al. found that the concentrations of glycerophospholipids and fatty acids in induced sputum are significantly lower in smokers with COPD than in smokers without COPD [61]. Cruickshank-Quinn et al. showed that glycerophospholipid metabolism in plasma is associated with worse airflow obstruction and more acute exacerbation in COPD [22]. Kelly et al. revealed that serum glycerophospholipids are associated with AHR, FEV1%, and FEV1/FVC% in asthmatic children, which indicates that the glycerophospholipid metabolism pathway plays a critical role in the pathogenesis of asthma [62]. Our study provides the first demonstration that glycerophospholipids in induced sputum are associated with lung function in patients with stable COPD. Therefore, the underlying mechanism of the glycerophospholipid metabolism pathway in COPD should be explored in future studies. Subsequently, multivariate analyses, namely, PCA and OPLS-DA, were used to observe the differences in the metabolomics profiles between moderate and severe COPD. Both the PCA and OPLS-DA results confirmed the significant separation between patients with moderate COPD and patients with severe COPD (Fig. 3). Collectively, these data indicate that the metabolomic profiles in induced sputum are associated with the severity of COPD.

Among the 21 MS2 metabolites (Supplementary Table S1), choline [63, 64], allopurinol [65, 66], o-xylene [67, 68], linoelaidyl carnitine [69, 70], vaccenyl carnitine [71, 72], pirimicarb [73, 74], and oxandrolone [75, 76] are involved in the oxidative stress response. It has been reported that the glycerophospholipid metabolism pathway plays an essential role in oxidative stress [22, 45–47, 77]. Therefore, the levels of five key oxidative stress products (MPO, SOD, GSH, NE, and 8-iso-PGF2α) in induced sputum were measured by ELISA in our study. We found that the levels of SOD, MPO, and 8-iso-PGF2α in patients with severe COPD were noticeably higher than those in patients with moderate COPD (Table 2). Moreover, negative correlations between SOD and three glycerophospholipids (choline, NMethy, and 1-LGPC) and between 1-LGPC and MPO and 8-iso-PGF2α were also detected (Table 3). Additionally, ROC curves were drawn to identify the values of the SOD, MPO, and 8-iso-PGF2α levels in induced sputum for predicting COPD severity. As shown in Fig. 4, the SOD, MPO, and 8-iso-PGF2α levels in induced sputum showed a good ability to predict the severity of stable COPD. Subsequently, we also identified significant negative correlations between FEV1% and the 8-iso-PGF2α levels and between FEV1/FVC% and the MPO and 8-iso-PGF2α levels (Table 3). Collectively, our results suggest that glycerophospholipid metabolism–associated oxidative stress is essential for the progression of COPD. The severity of oxidative stress is highly correlated with lung function impairments in patients with COPD. Therefore, the SOD, MPO, and 8-iso-PGF2α levels in induced sputum are promising markers for predicting the severity of COPD in clinical practice.

### Strengths and limitations

To the best of our knowledge, this study is the first to reveal that the association between the metabolomic profiles of induced sputum and COPD severity. The main strength of this study was that relatively comprehensive data, including demographic characteristics, underlying diseases, lung function, inhalation therapy, and laboratory parameters, were collected. Moreover, internal validity was good, which led to more convincing results. Of note, a chest HRCT scan was obtained from each patient, which enhanced the diagnosis accuracy and reduced the confounders. However, the limited sample size is one of the major weaknesses of this study. The roles of glycerophospholipid metabolism and its associated oxidative stress in COPD severity should be explored with a larger sample size. The predictive values of the glycerophospholipid and oxidative stress products (SOD, MPO, and 8-iso-PGF2α) levels for COPD severity should also be replicated in other populations and ethnic groups. Additionally, the alterations in COPD severity–associated differential metabolites and oxidative stress products cannot be comprehensively evaluated by a cross-sectional design. Thus, a cohort study should be performed to further explore the mechanisms underlying the metabolomics and oxidative stress changes in the progression of COPD.

## Conclusions and expert recommendations

Overall, our study provides the first identification of the COPD severity–associated metabolomic profiles of induced sputum. We also revealed the potential value of the induced sputum metabolomics for predicting the severity of COPD. In addition, a subset of COPD severity–associated metabolites was identified, and the data indicate that the glycerophospholipid metabolism pathway plays a hub role in the progression of COPD and is thus likely a potential therapeutic target in COPD. Our results also suggest that the levels of SOD, MPO, and 8-iso-PGF2α in induced sputum are potential biomarkers for predicting COPD severity. Collectively, our complex analysis of induced sputum at the metabolomics level not only provides confirmation of the association between the metabolomic profiles of induced sputum and COPD severity but also indicates the promising value of induced sputum metabolomics in the PPPM-based management of COPD. We hypothesized that induced sputum metabolites could be used to predict the progression, treatment response, and prognosis of COPD at an early stage, which would allow PPPM-based intervention in COPD.

We recommend the utilization of metabolomics in PPPM-associated studies of COPD. COPD is one of most common global diseases with high heterogeneity, and PPPM is a future trend for COPD management. Complicated metabolomic alterations are involved in different aspects of COPD. Metabolomics is a valuable and meaningful tool for the application of PPPM to COPD. Additionally, in future studies, the role of induced sputum metabolomics in the individual treatment response will be explored to further elucidate the value of metabolomics in the PPPM-based management of COPD. Specifically, based on individual metabolomic profiles, personalized management could be provided to patients with COPD.

## Electronic supplementary material

Table S1 (XLSX 2629 kb)

Table S2 (XLSX 129 kb)

## Abbreviations

1-LGPC: 1-linoleoylglycerophosphocholine
ACO: asthma-COPD overlap
ALI: acute lung injury
COPD: chronic obstructive pulmonary disease
CRP: C-reactive protein
EPA: eicosapentaenoic acid
ESR: erythrocyte sedimentation rate
FEV1%: forced expiratory volume in 1st second %
FVC: forced vital capacity
GOLD: Global Initiative for Chronic Obstructive Lung Disease
GSH: glutathione
HRCT: high-resolution CT
ILDs: interstitial lung diseases
IQR: interquartile range
KEGG: Kyoto Encyclopedia of Genes and Genomes
LC-MS/MS: liquid chromatography-mass spectrometry
mMRC: Modified Medical Research Council Dyspnea Scale
MPO: myeloperoxidase
MS: mass spectrometry
MS1: mass spectrometry 1
MS2: mass spectrometry 2
NCE: normalized collision energy
NE: neutrophil elastase
NEG: negative
NMethy: *N*-methylethanolamine phosphate
OPLS-DA: orthogonal projections to latent structures discriminant analysis
PCA: principal component analysis
POS: positive
PTE: pulmonary thromboembolism
QC: quality control
RT: room temperature
SD: standard deviation
SHS: suboptimal health status
SOD: superoxide dismutase
TB: tuberculosis
VIP: variable importance in the projection
